# Relationship between the neutrophil-to-lymphocyte ratio or platelet-to-lymphocyte ratio and deep venous thrombosis (DVT) following femoral neck fractures in the elderly

**DOI:** 10.3389/fsurg.2022.1001432

**Published:** 2022-10-13

**Authors:** Shuai Niu, Yueying Pei, Xin Hu, Dianzhu Ding, Guangwei Jiang

**Affiliations:** ^1^ Department of Vascular Surgery, The General Hospital of Hebei Province, Shijiazhuang, China; ^2^ Department of Doppler Ultrasonic, The General Hospital of Hebei Province, Shijiazhuang, China; ^3^ Department of Orthopaedic Surgery, The Third Hospital of Hebei Medical University, Shijiazhuang, China

**Keywords:** NLR, PLR, femoral neck fracture, elderly, DVT, clinical epidemiology

## Abstract

**Purpose:**

This study aimed to investigate whether the neutrophil-to-lymphocyte ratio (NLR) or platelet-to-lymphocyte ratio (PLR) was associated with deep venous thrombosis (DVT) following femoral neck fractures in the elderly.

**Method:**

This was a retrospective cohort study and used data extracted from the hospitalization electronic medical record and the laboratory biomarker reports. Patients were included if they were aged above 60 years with a definite diagnosis of femoral neck fracture caused by low-energy trauma. Duplex ultrasound scanning was routinely performed to detect the potential DVT. Two independent multivariate logistic regression models were constructed to identify the association of NLR or PLR with the risk of DVT.

**Results:**

A total of 708 patients with femoral neck fractures were included, and 112 were found to have DVT, indicating an incidence rate of 15.8%. There were significant differences across five subgroups for NLR or PLR, in terms of age (*p* = 0.020, 0.006), white blood cell (*p* < 0.001, =0.006), hemoglobin (*p* < 0.001, <0.001), and albumin (*p* < 0.001, <0.001). BMI was tested to be significantly different across subgroups for NLR (*p* = 0.030) and prevalence of cerebrovascular disease for PLR (*p* = 0.014). The multivariate analyses demonstrated that not NLR but PLR in Q3 (range, 179–238) was associated with an increased risk of DVT, and the risk for the latter was 1.86 (95%CI, 1.07–3.36).

**Conclusion:**

We concluded that a PLR value of 179–238 was associated with a 1.86-fold increased risk of DVT after femoral neck fracture. This study paves the way toward further exploration of inflammatory/immune biomarkers with the risk of DVT in the elderly with trauma.

## Introduction

As the population is aging, age-related fragile fractures have become an increasing healthcare issue, especially the hip fracture, notable as the “last fracture” in one's lifetime. It is projected that the number of hip fractures will rise to 6.26 million by 2050, with more than 50% occurring in Asia (1). Femoral neck fractures and intertrochanteric fractures are two typical patterns of hip fractures, with the latter one occurring at a more advantaged age, about 6–10 years older than the former one. Therefore, patients suffering intertrochanteric fractures would be at greater risk of postoperative systematic disorders and complications. Postinjury blood hypercoagulability, vascular endothelial injury, and long-term immobility would place the patients at a higher risk of deep venous thrombosis (DVT) or pulmonary embolism (PE).

Consequently, 10%–23.9% of patients experiencing hip fractures would die within 1 year, regardless of whether they underwent surgical treatment or not (2–4). Furthermore, 30%–60% of the survivors would experience a partial or complete loss of independence in daily activities, and 10%–20% are institutionalized following fractures (5). Differing from intertrochanteric fractures and subtrochanteric fractures, elderly patients with femoral neck fractures will be at an increased risk of perioperative complications and unfavorable prognosis, including avascular necrosis of femoral head (ANFH) that necessitates secondary hip joint replacement (6).

Among the perioperative complications following femoral neck fractures, deep venous thrombosis (DVT) is the most common one, involving 11%–40% of the fracture sufferers (7). Given its serious clinical consequence (pulmonary embolism and mortality), early identification of related factors and, accordingly, prompt patient-targeted intervention based on risk stratification results are of vital importance. By far, researchers have made multi-aspect attempts and identified some risk factors associated with DVT, including advanced age, obesity, history of DVT or pulmonary embolism, immobility of injured extremity, or the fracture itself (8–10). In the past decade, research studies on this topic have switched to the area of post-trauma inflammatory/immune, and the increasing evidence has suggested the role of easily available systemic inflammatory/immune biomarkers in the outcome prediction of a variety of medical conditions, including ischemic stroke (11, 12), cerebral hemorrhage (13, 14), and major cardiac events (15, 16). In addition, in major orthopedics surgery fields, researchers have identified significant associations between the neutrophil-to-lymphocyte ratio (NLR) and platelet-to-lymphocyte ratio (PLR) with acute DVT (17, 18). However, this relationship has not always been consistently demonstrated (19, 20).

Considering the potential adverse effect of DVT in elderly patients and the nature of low-cost and ready accessibility of these inflammatory/immune biomarkers, it is necessary to identify their relationship with DVT. To the best of our knowledge, no studies are specifically aimed at addressing this issue. In this study, we aimed to identify whether there is an association between NLR or PLR and the risk of DVT occurring after admission, after adjustment for demographic characteristics, comorbidities, and other laboratory variables.

## Materials and methods

### Inclusion and exclusion criteria

This retrospective study was approved by the ethics committees of the Hebei General Hospital, which waived the requirement for informed consent due to the deidentification of data used. The methods were performed in accordance with the Helsinki Declaration and following the Strengthening the Reporting of Cohort Studies in Surgery (STROCSS). Between January 2016 and December 2020, patients 60 years or older who had acute femoral neck fractures caused by low-energy mechanism (fall from a standing height) in the two participating territory hospitals (The Third Hospital of Hebei Medical University and Hebei General Hospital) were initially included. The exclusions criteria are as follows: pathological (metastatic) fracture, time to admission above 1 week, fracture caused by high-energy mechanism, open fracture, concurrent fracture at other locations, vascular injury of other organs, history of abnormal lower limb muscle strength, history of DVT or other thrombotic events, thrombophilia or hematological disorders, and current use of anticoagulants or glucocorticoids (within 3 months of injury) due to chronic comorbidities.

### Definition and management of DVT

DVT was diagnosed using the guidelines for the diagnosis of DVT proposed by the Chinese Medical Association (3rd edition) (21). According to the institutional protocol, duplex ultrasound (DUS) scanning of the deep veins of the bilateral lower extremities for detection of potential thrombosis is compulsory for elderly patients with femoral neck fractures after they are admitted for hospitalization. The scanned veins include common femoral, superficial femoral, deep femoral, popliteal, posterior tibial, anterior tibial, peroneal veins, and intermuscular venous plexus of the calf. The diagnostic criteria for DVT were loss of noncompressibility of the scanned vein, lumen obstruction or filling defect, lack of respiratory variation in above-knee vein segments, and inadequate flow augmentation to the calf and foot with compression maneuvers.

Based on the findings of DUS scanning, prophylactic or therapeutic chemical thromboprophylaxis was administered, including subcutaneous injection of low-molecular-weight heparin (LMWH). Physical prophylaxis included elevation of injured extremity, quadriceps strength exercise, and ankle pump practices were routinely administered for each patient since admission.

### Data collection

Routine blood tests were performed in accordance with the manufacturer-recommended methods by using a hematology analyzer (UniCel DxH 800; Beckman Coulter, Brea, CA, USA) for complete blood count test and an autoanalyzer AU5800 (Beckman Coulter, United States) for biochemical tests. Data were extracted from the patients’ hospitalization electronic medical records and the laboratory biomarker reports. Biomarkers or biomarker-derived inflammatory/immune indexes at admission or the first time after admission were selected for data analysis to eliminate their potential time-dependent effect to the maximum extent.

Data of interest included neutrophil count, lymphocyte count, platelet count, NLR defined as neutrophil count divided by lymphocyte count, and PLR defined as platelet count divided by lymphocyte count. Given its clinical value in predicting or auxiliary diagnosing DVT, plasma D-dimer level was included. Albumin (ALB) level, as an important nutritional index or inflammatory index, and hemoglobin (HGB) level, as an index reflecting anemia status or invisible bleeding due to the index femoral neck fracture, were also included. Other potential factors for adjustment included demographics [age, gender, body mass index (BMI)], smoking, alcohol abuse, and comorbidities (hypertension, diabetes, chronic heart disease, cerebrovascular disease, pulmonary disease).

The comorbidities documented in the medical records were identified in accordance with the discharge diagnosis based on patients’ self-reports with or without physical or laboratory examination. For statistical analysis purposes, we collectively refer to chronic heart failure, arrhythmias, myocardial ischemia diseases, coronary artery disease, and valvular heart disease as “cardiac diseases”; a history of stroke, cerebral thrombosis, cerebral hematoma, cerebral hemorrhage, hemiplegia, hemiparesis or carotid artery stent or angioplasty as cerebrovascular disease; and chronic obstructive pulmonary disease, emphysema, chronic bronchitis, cystic fibrosis or asthma as pulmonary disease.

### Statistical analysis

Continuous variables were presented with mean ± standard deviation (SD) or median and interquartile range (IQR) based on the normality status determined by the Shapiro–Wilks normality test, and between-group difference was evaluated by Student’s *t*-test (normal variable) or the Mann–Whitney *U* test (skewed variable) for two groups by one-way analysis of variance (ANOVA) (normal variable) or the Kruskal–Wallis *H* test (skewed variable) for multiple groups. Categorical variables were presented as count and percentage, and the between-group differences were evaluated by the chi-square or Fisher's exact test, as appropriate.

First, the multivariate analysis using a linear regression model was used to investigate the relationship between NLR and PLR with the baseline variables. Second, NLR and PLR were divided into five subgroups based on their baseline data, including quartiles and an extra subgroup with a value above the 95^th^ percentile. As for NLR, the five subgroups were quartile 1, <3.62; quartile 2, 3.62–5.54; quartile 3, 5.55–8.48; quartile 4, >8.48; and >95^th^ percentile (>15.9). As for PLR, the five subgroups were quartile 1, <131; quartile 2, 131–178; quartile 3, 179–238; quartile 4, >238; and >95^th^ percentile, >410. For NLR or PLR, comparisons among five subgroups for continuous variables (age, BMI, ALB, HGB, WBC, plasma D-dimer level) were performed using ANOVA and for categorical variables (gender and prevalence of each comorbidity) using the chi-square or Fisher's exact test.

Considering the significantly positive correlation between NLR and PLR (Pearson’s correlation coefficient, 0.696; *p* < 0.001), two independent multivariate logistic regression models, for NLR or PLR, respectively, were constructed to estimate the odd ratio (OR) with 95% confidence interval (95%CI) for DVT across the five subgroups of NLR or PLR using quartile 1 as reference. The analyses were adjusted for demographics, comorbidities, and other laboratory biomarkers mentioned above. *p* values less than 0.05 were considered statistically significant. All analyses were performed using SPSS 23.0 (IBM, Armonk, New York, USA).

## Results

**Figure 1:**
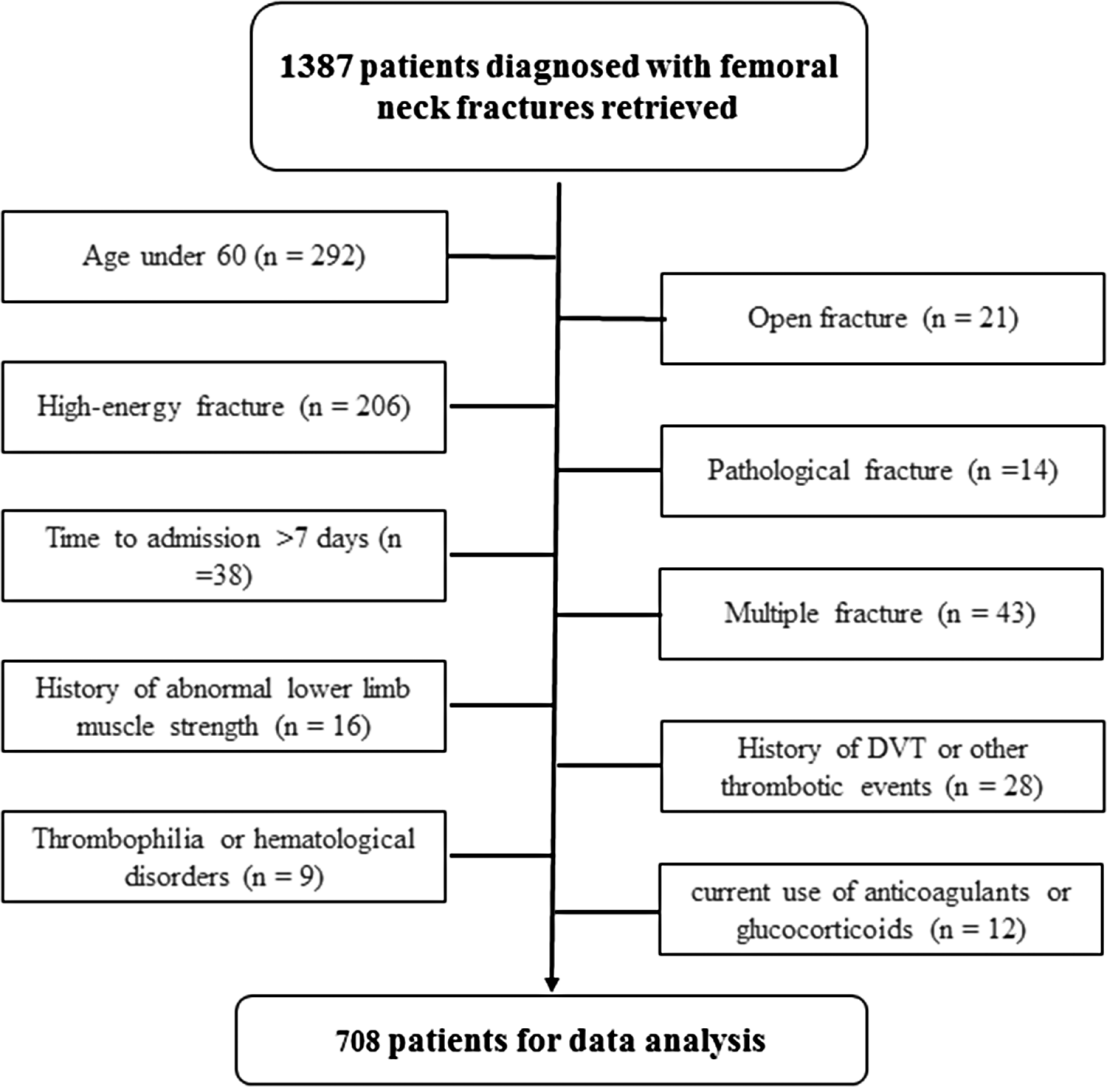
Flowchart showing the inclusion and exclusion of subjects.

During the study period, there were 1,387 patients presenting with femoral neck fractures, and based on our criteria, 679 were excluded, leaving 708 for data analysis (Figure 1). Among these 708 patients, there were 212 males and 496 females, and their mean age was 71.7 ± 8.2 years (range, 60–96 years). One hundred and twelve patients were found to have developed a DVT after admission, indicating an incidence rate of 15.8% (13.1%–18.5%). The time from injury to laboratory assessment was 1.4 ± 0.5 days, ranging from 0 to 3 days. The time from injury to detection of DVT was 2.7 ± 3.1 days. Patients with DVT had a significantly higher value of platelet count (231.0 ± 71.1 vs. 208.5 ± 79.2, *p* = 0.005) and lymphocyte count (1.3 ± 0.4 vs. 1.2 ± 0.5, *p* = 0.027), a lower value of NLR (5.7 ± 3.0 vs. 6.9 ± 4.8, *p* = 0.009), and a trend to a lower serum albumin level (33.8 ± 5.7 vs. 34.9 ± 5.8, *p* = 0.068), compared to those without DVT. However, in terms of demographics (age, 71.1 ± 7.4 vs. 71.9 ± 8.3; proportion of male patients, 30.4% vs. 29.9%), prevalence of comorbidities (hypertension, 52.7% vs. 48.3%; diabetes, 21.4% vs. 22.0%; history of a cerebrovascular accident, 22.3% vs. 26.7%, cardiac disease, 16.1% vs. 22.1%; pulmonary disease, 0.9% vs. 3.0%), and other biomarkers, there was no significant difference for either one (all *p*’s > 0.05). Over 50% of patients (371, 52.4%) were treated by total hip arthroplasty, 127 (17.9%) by hemiarthroplasty, and 210 (29.7%) by osteosynthesis procedure.

The mean NLR value was 6.7 (SD, 4.5), ranging from 0.7 to 28.1. Among the DVT group, the mean NLR value was 5.7 (SD, 3.0), significantly lower than that of the non-DVT group (mean, 6.9; SD, 4.8), with a *p* value of 0.010. The multivariate linear regression analysis showed that female sex [standard coefficient (*β*), −0.113; *p* = 0.001], WBC (*β*, 0.485; *p* < 0.001), and serum albumin (*β*, −0.078; *p *= 0.034) were significantly associated with NLR. Among the five subgroups, a significant difference was found between DVT and non-DVT groups for Q5 (18.3 ± 1.2 vs. 21.3 ± 3.8, *p* = 0.006) but not for Q1 (2.3 ± 0.7 vs. 2.5 ± 0.7, *p* = 0.161), Q2 (4.5 ± 0.5 vs. 4.7 ± 0.6, *p* = 0.057), Q3 (7.1 ± 0.9 vs. 6.9 ± 0.9, *p* = 0.484), or Q4 (10.4 ± 1.5 vs. 11.0 ± 1.9, *p* = 0.177). Age was found to be significantly different across five subgroups (*p* = 0.020), with a linear trend toward older age with higher levels of subgroups. In terms of BMI, WBC, HGB, and ALB, we observed significant differences across the five subgroups (all *p* values <0.05). For HGB and ALB, a downward trend was observed with higher NLR quartiles. However, regarding other variables, such as gender, smoking, alcohol abuse, and any chronic comorbidity, we did not observe a statistically significant difference (all *p* values above 0.05) (Table 1).

**Table 1:** Comparisons of baseline characteristics of included patients across five subgroups of NLR.

NLR	Q1	Q2	Q3	Q4	>95th percentile	*p*
	<3.62	3.62–5.54	5.55–8.48	>8.48	>15.9	
Age (years)	71.7 ± 8.1	71.0 ± 8.4	71.1 ± 8.2	72.7 ± 7.8	75.6 ± 8.0	0.020
Gender (male, %)	45 (25.4)	50 (28.2)	53 (30.1)	64 (40.0)	12 (35.3)	0.283
BMI (kg/m^2^)	22.8 (20.2, 25.5)	24.0 (21.4, 25.7)	24.0 (22.0, 26.0)	23.4 (21.3, 25.7)	23.0 (19.5, 25.8)	0.037^a^
Smoking (%)	19 (10.7)	21 (11.8)	16 (9.0)	22 (12.4)	5 (14.7)	0.757
Alcohol abuse (%)	23 (13.0)	27 (15.2)	26 (14.7)	30 (16.9)	7 (20.6)	0.802
Hypertension (%)	81 (45.8)	84 (47.5%)	88 (50.0)	94 (52.8)	20 (58.8)	0.623
Diabetes (%)	37 (20.9)	34 (19.2)	48 (27.3)	30 (16.9.)	6 (17.6)	0.370
Cerebrovascular disease (%)	54 (30.5)	38 (21.5)	47 (26.7)	45 (25.3)	12 (35.3)	0.196
Heart disease (%)	33 (18.6)	41 (23.2)	37 (21.0)	39 (21.9)	10 (29.4)	0.631
Pulmonary disease (%)	7 (4.0)	9 (5.1)	7 (4.0)	8 (4.5)	3 (8.8)	0.317
WBC (*10^9^/L)	6.5 (5.3, 7.6)	8.1 (6.7, 9.5)	8.9 (7.5, 10.4)	9.9 (8.7, 11.8)	11.5 (8.8, 14.3)	<0.001^a^
HGB (g/L)	119.9 ± 15.2	119.2 ± 16.9	114.7 ± 17.1	113.4 ± 15.6	110.3 ± 15.7	<0.001
ALB (g/L)	35.8 ± 5.8	35.8 ± 5.5	34.7 ± 5.6	33.0 ± 5.7	31.7 ± 6.9	<0.001
D-dimer	0.59 (0.23, 1.16)	0.9 (0.42, 1.99)	0.93 (0.42, 2.87)	0.83 (0.44, 1.56)	0.64 (0.34, 1.47)	0.231^a^

NLR, neutrophil-to-lymphocyte ratio; Q, quartile; BMI, body mass index; WBC, white blood cell; HGB, hemoglobin; ALB, albumin.

^a^ Mann–Whitney *U* test.

The mean PLR was 200 (SD, 109), ranging from 24 to 1005. Among the DVT group, the mean PLR value was 189.8 (SD, 71.3), significantly lower than that of the non-DVT group (mean, 202.2; SD, 114.6), with a *p* value of 0.039. As the same, female sex, WBC, and serum albumin were significantly associated with PLR, with *β* values of −0.082, 0.092, and −0.097 and *p* values of 0.034, 0.013, and 0.022, respectively. Additionally, age tends to correlate positively with PLR, with a *β* of 0.068 and a *p* value of 0.074. Among the five subgroups, a significant difference was found between DVT and non-DVT groups for Q3 (212.7 ± 18.4 vs. 204.9 ± 17.1, *p* = 0.017), Q4 (269.1 ± 43.8, *p* = 0.017), and Q5 (421.7 ± 8.1 vs. 549.7 ± 121.6, *p* < 0.001) but not for Q1 (103.7 ± 16.5 vs. 98.7 ± 22.6, *p* = 0.318) and Q2 (152.4 ± 14.0 vs. 154.4 ± 12.9, *p* = 0.426). There were significant differences among five subgroups in terms of age (*p* = 0.006), WBC (*p* = 0.006), HGB (<0.001), and ALB (*p* < 0.001) (Table 2). However, as for any other variables, no significant difference was found (all *p* values > 0.05) (Table 2).

**Table 2:** Comparisons of baseline characteristics of included patients across five subgroups of PLR.

PLR	Q1	Q2	Q3	Q4	>95th percentile	*p*
	<131	131 to 178	179 to 238	>238	>410	
Age (years)	71.6 ± 8.3	71.2 ± 8.0	70.8 ± 7.9	73.0 ± 8.3	75.5 ± 7.9	0.006
Gender (male, %)	50 (28.4)	62 (35.2)	49 (27.8)	51 (28.7)	13 (37.1)	0.315
BMI (kg/m^2^)	23.4 (20.9, 26.2)	23.4 (21.5, 25.7)	23.9 (21.3, 25.7)	23.4 (20.8, 26.3)	22.5 (19.5, 25.4)	0.177^a^
Smoking (%)	16 (9.0)	19 (10.7)	23 (12.9)	20 (11.2)	4 (11.8)	0.759
Alcohol abuse (%)	25 (14.1)	25 (14.1)	29 (16.4)	27 (15.2)	6 (17.6)	0.782
Hypertension (%)	81 (46.0)	78 (44.3)	88 (50.0)	100 (56.2)	20 (57.1)	0.251
Diabetes (%)	32 (18.2)	35 (19.9)	48 (27.3)	40 (22.5)	9 (25.7)	0.274
Cerebrovascular disease (%)	43 (24.4)	58 (33.0)	34 (19.3)	49 (27.5)	14 (40.0)	0.014
Heart disease (%)	42 (23.9)	32 (18.2)	34 (19.3)	42 (23.6)	8 (22.9)	0.631
Pulmonary disease (%)	9 (5.1)	5 (2.8)	6 (3.4)	11 (6.2)	3 (8.8)	0.262
WBC (*10^9^/L)	8.0 (6.4, 9.5)	8.2 (6.8, 10.4)	8.1 (6.9, 9.8)	8.9 (7.4, 10.8)	9.5 (7.3, 13.2)	0.011^a^
HGB (g/L)	121.0 ± 15.7	120.2 ± 16.5	114.2 ± 16.6	111.4 ± 15.1	110.5 ± 14.7	<0.001
ALB (g/L)	35.9 ± 5.7	35.8 ± 5.5	34.4 ± 5.7	33.1 ± 6.1	32.2 ± 5.2	<0.001
D-dimer	0.78 (0.32, 1.80)	0.80 (0.41, 2.00)	0.82 (0.39, 2.22)	0.84 (0.43, 1.74)	0.62 (0.31, 1.00)	0.172^a^

PLR, platelet-to-lymphocyte ratio; Q, quartile; BMI, body mass index; WBC, white blood cell; HGB, hemoglobin; ALB, albumin.

^a^ Mann–Whitney *U* test.

Table 3 lists the ORs and adjusted ORs for the development of DVT in different subgroups of NLR or PLR, with quartile 1 set as the reference. In univariate analysis, only quartile 3 of baseline PLR showed a significant association with increased risk of DVT (OR, 1.05–3.31). For any other comparisons, for either NLR or PLR, the association with the risk of DVT was not statistically significant. As the same, the multivariate analyses demonstrated a nonsignificant association with the development of DVT for subgroups, except for Q3 (OR, 1.86; 95%CI, 1.07–3.36) compared to Q1 for PLR.

**Table 3:** Incidence rate of DVT following femoral neck fracture across the five subgroups for NLR or PLR and the crude and adjusted ORs.

Item	DVT cases	Incidence rate (%)	Crude OR (95%CI)	Adjusted OR (95%CI)^a^	*p*
NLR					
Q1	28	15.8	Reference	Reference	
Q2	33	18.6	1.22 (0.70–2.12)	1.31 (0.74–2.33)	0.493
Q3	26	14.7	0.92 (0.61–1.95)	0.8 (0.4–1.5)	0.384
Q4	25	14.0	0.76 (0.41–1.38)	0.7 (0.3–1.5)	0.302
>95th percentile	3	8.8	0.51 (0.15–1.80)	0.4 (0.2–1.4)	0.875
PLR					
Q1	22	12.4	Reference	Reference	
Q2	31	17.5	1.50 (0.83–2.70)	1.61 (0.88–2.94)	0.161
Q3	37	20.9	1.86 (1.05–3.31)	1.86 (1.07–3.36)	0.044
Q4	22	12.4	1.00 (0.53–1.88)	1.00 (0.51–1.98)	0.880
>95th percentile	3	8.8	0.68 (0.19–2.42)	0.62 (0.17–2.29)	0.475

DVT, deep venous thrombosis; NLR, neutrophil-to-lymphocyte ratio; PLR, platelet-to-lymphocyte ratio; OR, odd ratio.

^a^ Adjusted for the following covariates at baseline in the logistics regression model: age, gender, body mass index, smoking, alcohol abuse, diabetes, hypertension, cerebrovascular disease, heart disease, pulmonary disease, hemoglobin, albumin, white blood cell, and plasma D-dimer level.

## Discussion

Recently, emerging evidence has suggested that there is some association between inflammatory/immune indexes (NLR or PLR) and adverse events, i.e., early deaths or myocardial infarction events after coronary artery disease (22, 23), deaths after various cancers (24), venous thromboembolisms (VTEs) after arthroplasty surgeries (11), postoperative mortality and cardiovascular complications after hip fracture surgery (25, 26), and reduced bone mineral density in the elderly population (27). In this study, we focused on a specific subgroup of elderly patients who experienced femoral neck fractures but failed to demonstrate the association between admission NLR or PLR and the risk of DVT.

NLR and PLR are simple and readily available hematology panel biomarkers of inflammatory/immune, and reference intervals of their values in the population have been well established. Meng et al. (28) retrospectively evaluated the data of a cohort of 24,029 healthy physical examinees in Henan, China, and reported that the NLR was 1.72 for males and 1.71 for females, while the PLR was 102 for males and 115 for females. Furthermore, the authors found a significantly higher NLR value in the elderly (≥65 years) than in the younger (18–65 years) population, as well as a similar trend for PLR. In another study including more than 380,000 adults in Wuhan, China, the authors reported similar results for NLR (males, 1.67; females, 1.68) (29). Specifying the field of trauma, Alexandru et al. (30) found a higher NLR (4 to 5.6) or PLR (99 to 126.4) in patients with either humeral, femoral, or tibial diaphyseal fracture than the controls, and the NLR relative to PLR was a more sensitive biomarker. In this study, we found higher NLR and PLR values in elderly patients with femoral neck fractures, which were about 3.6-fold and 1.4-fold of those reported above (28, 29). We believe that our findings reflect the impacts of both the fracture and advanced age on the inflammation/immune status.

In various subspecialties of orthopedics, the association between NLR or/and PLR and VTEs has been investigated in the literature, although the conclusion was not always consistent. In a matched case–control study, Barker et al. (11) found that the NLR increase was greater following total knee arthroplasty (TKA) than that following unicompartmental knee arthroplasty (UKA), which indicated that the degree of surgical trauma varied as did the effect on the inflammation/immune reaction. Additionally, the authors demonstrated the significant association of NLR with VTE and suggested NLR as a matrix to identify whether a patient is susceptible to developing VTE after TKA. Yao et al. (12) got a similar conclusion in their study of 773 cases of total joint arthroplasty, although they concluded that NLR or PLR could not predict DVT accurately due to the low specificity and sensitivity. In the specific study of traumatic fractures, including one recent study of tibial plateau fractures (20), no significant association has not been investigated. By far as we know, this was the first study aiming to investigate the NLR or PLR as risk factors for DVT following femoral neck fractures. Although the growing evidence supported the NLR or PLR associated with DVT, we failed to get that conclusion in this specific subgroup, except for the Q3 of PLR (range, 179–238; OR = 1.86 and 95%CI = 1.07–3.36). The detailed mechanism underlying this has not been well explored, and future studies should be focused on the innate immune responses to trauma and the dynamic change of inflammatory/immune indexes after trauma (31). Another research focus should be directed at characterizing the inflammatory/immune or stress to trauma in the elderly, which is speculated to be different from those of younger age.

The strengths of this study were including a large sample of participants and investigating the relationship between the novel biomarker parameters and preoperative DVT after femoral neck fractures in elderly patients. This study has some limitations. First, the retrospective nature was its inherent limitation, which might not allow absolute accuracy in data collection. Also, patients’ recall bias or unawareness of the already existing comorbidities may compromise the reliability of the conclusion. Second, as with every other multivariate analysis, not all potential factors influencing the occurrence of DVT could be taken into consideration, which left the confounding effects remaining. For example, the fracture severity might have an impact on the coagulation state but was not considered before the commencement of this study. Third, C-reactive protein (CRP) is an important inflammatory biomarker but could be found in only a small proportion (17.5%) of patients. Fourth, we identified association rather than causality, and therefore, these results should be interpreted with caution. Fifth, this study was conducted in two tertiary referral hospitals, which may be at risk of selection bias because patients treated in these centers are more likely to be medically complex. As such, the generalizability of the results to others may be affected to some extent.

In conclusion, the incidence rate of DVT following femoral neck fracture in the elderly was 15.8%. A PLR value of 179–238 was associated with a 1.86-fold increased risk of DVT, while NLR was not. These findings added knowledge to the literature by introducing the two novel biomarkers to a specific elderly population with hip fractures. Future studies need to identify the physiopathologic mechanism of PLR in relation to DVT and its potential role in the prognosis.

## Data Availability

The original contributions presented in the study are included in the article/Supplementary Material, further inquiries can be directed to the corresponding author/s.
